# Theories of change for e-health interventions targeting HIV/STIs and sexual risk, substance use and mental ill health amongst men who have sex with men: systematic review and synthesis

**DOI:** 10.1186/s13643-020-01523-2

**Published:** 2021-01-11

**Authors:** Rebecca Meiksin, G. J. Melendez-Torres, Jane Falconer, T. Charles Witzel, Peter Weatherburn, Chris Bonell

**Affiliations:** 1 London School of Hygiene & Tropical Medicine, 15-17 Tavistock Place, London, WC1H 9SH UK; 2 South Cloisters, University of Exeter, St Luke’s Campus, Heavitree Road, Exeter, EX1 2 LU UK

**Keywords:** e-Health, Digital health, Men who have sex with men, Sexual health, HIV, STI, Substance use, Mental health, Systematic review, Theory of change

## Abstract

**Background:**

Sexual risk, substance use, and mental ill health constitute a syndemic of co-occurring, mutually reinforcing epidemics amongst men who have sex with men (MSM). Developed since 1995, e-health interventions offer accessible, anonymous support and can be effective in addressing these outcomes, suggesting the potential value of developing e-health interventions that address these simultaneously amongst MSM. We conducted a systematic review of e-health interventions addressing one or more of these outcomes amongst MSM and in this paper describe the theories of change underpinning relevant interventions, what these offer and how they might complement each other.

**Methods:**

We identified eligible reports via expert requests, reference-checking and database and Google searches. Results were screened for reports published in 1995 or later; focused on MSM; reporting on e-health interventions providing ongoing support to prevent HIV/STIs, sexual risk behaviour, substance use, anxiety or depression; and describing intervention theories of change. Reviewers assessed report quality, extracted intervention and theory of change data, and developed a novel method of synthesis using diagrammatic representations of theories of change.

**Results:**

Thirty-three reports on 22 intervention theories of change were included, largely of low/medium-quality. Inductively grouping these theories according to their core constructs, we identified three distinct groupings of theorised pathways. In the largest, the ‘cognitive/skills’ grouping, interventions provide information and activities which are theorised to influence behaviour via motivation/intention and self-efficacy/perceived control. In the ‘self-monitoring’ grouping, interventions are theorised to trigger reflection, self-reward/critique and self-regulation. In the ‘cognitive therapy’ grouping, the theory of change is rooted in cognitive therapy techniques, aiming to reframe negative emotions to improve mental health.

**Conclusions:**

The synthesised theories of change provide a framework for developing e-health interventions that might holistically address syndemic health problems amongst MSM. Improving reporting on theories of change in primary studies of e-health interventions would enable a better understanding of how they are intended to work and the evidence supporting this. The novel diagrammatic method of theory of change synthesis used here could be used for future reviews where interventions are driven by existing well-defined behaviour and behaviour change theories.

**Systematic review registration:**

PROSPERO CRD42018110317

**Supplementary Information:**

The online version contains supplementary material available at 10.1186/s13643-020-01523-2.

## Introduction

Despite major advances in treatments and pharmacological prevention, men who have sex with men (MSM) continue to experience increased risk of infection with HIV and other sexually transmitted infections (STIs) [1] and high levels of sexual risk behaviour [2]. MSM also report high rates of alcohol [3–5] and legal and illegal drug use (henceforth referred to as substance use) [6–8] and of common markers of mental ill health [9]. MSM are twice as likely to be depressed or anxious compared to other men [10]. Nationally representative surveys in the UK suggest that almost half of MSM experience one or more of these outcomes [11].

Sexual risk, substance use and mental ill health are increasingly considered to constitute a syndemic of simultaneous, mutually reinforcing epidemics. There is consistent evidence that these outcomes can inter-correlate strongly at the level of the individual and the sexual event [12–18]. Drug use can be both a symptom and cause of mental ill health, and both drug use and mental ill health can increase sexual risk behaviours [19, 20]. MSM who report using certain drugs, such as nitrite inhalants, and ketamine and other drugs linked to sex parties, are more likely to report sexual risk with multiple partners [21]. Survey data indicate that MSM reporting substance use are more likely to report unprotected anal intercourse and HIV infection [22]; MSM with higher levels of anxiety and depression are more likely to have potential alcohol dependency [2]; and MSM with depressive symptoms report more condomless anal sex [23]. Public health strategies to address these outcomes together therefore have the potential to achieve multiplicative effects.

Stigma and discrimination pose barriers to appropriate services for MSM worldwide [24–26]. Online global surveys of MSM have found that only 11% of substance-using participants reported accessible treatment programmes, with only 5% reporting use [27], and that fewer than half of participants had easy access to condoms and HIV testing [28]. A study of MSM attending UK sexual health clinics found that 42% of those with depressive symptoms were not diagnosed and 48% were not receiving treatment [29]. There is an urgent need for accessible and effective new strategies to address these outcomes amongst MSM.

e-Health interventions, facilitated via mobile phones, internet or other electronic communication technology, can be delivered in a variety of formats such as text messaging, smartphone/mobile apps, games and other online content. e-Health interventions aim to promote healthy behaviours and mental health, for example, by setting and reviewing goals; increasing/maintaining motivation; providing feedback on behaviour and challenging thought patterns that obstruct change. There is good evidence from systematic reviews focused on general or mixed populations that e-health interventions can reduce alcohol use [30] and address common causes of mental ill health [31–37]. Emerging evidence also suggests that e-health interventions might reduce drug use and sexual risk behaviour [38–41]. Given the clustered and interacting nature of these problems amongst MSM, this might suggest the value of developing e-health interventions for MSM that addresses these outcomes simultaneously and holistically. Such an approach might well have multiplicative effects. However, existing mental health [42] and HIV prevention [43] e-health interventions rarely target MSM, and those that are not designed for MSM risk unacceptability or failure to address the specific needs of this population [42].

We therefore conducted a systematic review of e-health interventions addressing sexual health, substance use and/or mental health amongst MSM to explore the approaches and theories of how the interventions were intended to work to improve targeted health outcomes (“theories of change”) employed; factors promoting or impeding delivery or receipt of such interventions; their effectiveness in improving health outcomes and whether such interventions are cost-effective. We reviewed relevant interventions that targeted our three health outcomes of interest separately or together to maximise learning about what interventions exist that might be harnessed to address this syndemic and what the evidence from interventions addressing some but not all of these outcomes suggest could be developed to target all three holistically.

In this paper, we report findings from our synthesis of intervention theories of change to understand how these interventions are intended to work. Intervention theories of change typically draw on existing scientific theories of behaviour (which consider factors that predict behaviours) and/or existing scientific theories of behaviour change (which propose general mechanisms of changing behaviour). Intervention theories of change make explicit the hypothesised mechanisms through which intervention activities are intended to generate outcomes [44], helping developers to systematically consider and describe which psychological, social or other factors interventions should address to achieve their intended outcomes. They also help evaluators determine what to measure to assess whether and how an intervention works and which components, if any, are most important [44]. Review evidence suggests that increased use of scientific theory is associated with greater effectiveness of internet-based health behaviour change interventions [45], but intervention theories of change must take into account which mechanisms sustain the targeted outcomes in a particular context, amongst a particular population [46]. Our aim is to describe the range of intervention theories of change that have informed e-health interventions, what these offers and how they might complement each other, which has not been done in previous reviews addressing our outcomes of interest [39, 41, 42, 47, 48]. We aim to answer the following questions:

1. What theories of change inform e-health interventions addressing HIV/STIs, sexual risk behaviour, substance use or common mental illness symptoms amongst MSM?
2. What overarching theories of change are suggested by the theories of change used for included interventions?

## Methods

### Search strategy

Our methods were guided by a protocol prospectively registered on PROSPERO in September 2018 (registration number CRD42018110317) [49].

In this paper, we report what intervention theories of change were used in eligible studies that were retrieved as part of a broader systematic review. Reports eligible for the broader review were published in 1995 or later (because e-health interventions were unavailable prior to this); focused on gay, bisexual and other men (including cisgender and transgender men) who have sex with men; and reported on e-health interventions delivered via mobile phone, internet or other electronic communication technology that aimed to provide ongoing support to populations consisting entirely or principally of MSM to prevent HIV, STIs, sexual risk behaviour, alcohol and drug use or common causes of mental ill health (anxiety or depression). We excluded e-health interventions merely facilitating one-off as opposed to ongoing support, those addressing HIV self-testing, clinic attendance or STI partner notification only, and interventions delivered by human providers via electronic media. There were no limitations by language or geographical location of studies. Reports eligible for inclusion in the theory of change synthesis described intervention theories of change, logic models or theorised mechanisms of effect.

Our search strategy, refined after piloting, covered two core concepts: men who have sex with men; and e-health. Publication date was limited from 1995 to now and no language limits or limits by study methodology were added. We searched 19 health and social science bibliographic databases, 23 October-26 November 2018. These were ProQuest Applied Social Sciences Index & Abstracts (ASSIA) (1987-current); Campbell Library (complete database); CRD Databases (complete databases); EBSCO CINAHL Plus (complete database); Wiley Cochrane Library (complete database); EPPI-Centre Database of Health Promotion Research (Bibliomap) (complete database); ProQuest Dissertations & Theses Global (1951-current); OvidSP EconLit (1886 to October 18, 2018); OvidSP Embase (1980 to 2018 week 43); OvidSP Global Health (1910 to 2018 week 41); OvidSP Health Management Information Consortium (HMIC) (1979 to July 2018); ProQuest IBSS (1951-current); Ovid MEDLINE(R) and Epub Ahead of Print, In-Process & Other Non-Indexed Citations and Daily (1946 to October 22, 2018); OvidSP PsycInfo (1806 to October 2018 week 3,); Web of Science Science Citation Index Expanded (1970-present, data last updated 24 October 2018); Elsevier Scopus (complete database); OvidSP Social Policy & Practice (201807); Web of Science Social Sciences Citation Index Expanded (1970-present, data last updated 24 October 2018); ProQuest Sociological Abstracts (1952-current as of 29 October 2018). Full details of the search strings for each are available at the London School of Hygiene & Tropical Medicine’s Data Repository [50] and full details of the search conducted in the OvidSP Medline database are available in Additional file 1. We also searched three clinical trials registers for relevant ongoing and unpublished studies (ClinicalTrials.gov; the World Health Organisation International Clinical Trials Registry Platform; the EPPI-Centre Trials Register of Promoting Health Interventions); Google for non-governmental organisation and governmental publications (limited to the first 100 results); the OpenGrey database; and references of included reports. We contacted experts to request eligible reports. Citations were uploaded to EndNote and de-duplicated. Database and trials register searches were updated 22-27 April 2020.

### Screening and data extraction

Two reviewers piloted an inclusion criteria worksheet by screening batches of the same 50 references, resolving disagreements by consensus. After piloting achieved an agreement rate of at least 95%, each reference was screened on title and abstract by one reviewer. A second round of screening with a comparable piloting process then focused on full study reports. Where reports provided insufficient information on the intervention to determine eligibility, we contacted study authors. Two reviewers independently extracted data from each eligible report on intervention theory of change constructs and mechanisms, the evidence presented in support of the theory of change and how the theory of change was developed. We extracted data on intervention theory of change as well as the existing scientific theories of behaviour and behaviour change that informed these.

### Quality assessment

Two reviewers independently assessed the quality of each report included in the theory of change synthesis (i.e. each “theory report”) using a tool used in our previous systematic reviews [51, 52], modified to include assessment of whether intervention mechanisms were theorised as operating differently by context [53]. Quality assessment focused on (a) the extent to which the theory of change described the path from intervention to outcomes; (b) the clarity with which theoretical constructs were defined; (c) the clarity with which causal inter-relationships between constructs were defined; (d) the extent to which the mechanisms underlying these inter-relationships were explained and (e) the extent to which the theory of change considered how mechanisms and resultant outcomes might vary by context. The two reviewers met to compare their assessments, resolving any differences through discussion. Rather than restricting the synthesis to studies judged to be of high quality, conclusions drawing on poorer quality reports were given less interpretive weight.

### Synthesis of intervention theories

We undertook synthesis of author narratives describing theories of how interventions were intended to generate outcomes. We aimed first to summarise theories of change for specific interventions and then to examine whether there were one or more overarching theories of change relevant across different interventions. Theory synthesis commonly uses a meta-ethnographic approach, originally developed to synthesise findings across multiple qualitative studies [54], and now applied to theory synthesis. As originally applied to qualitative research, meta-ethnographic methods draw on primary constructs (verbatim qualitative data presented in reports of primary research) and secondary constructs (author interpretations of data presented in primary research) to develop tertiary constructs (reviewer interpretations presented in syntheses). Applied to theory synthesis, such methods draw solely on primary constructs (author descriptions of theory of change).

We initially planned to undertake line-by-line coding of theory reports in order to identify narrative themes within, and common themes across, intervention theories of change as we have done in previous theory syntheses [51, 52]. In the first stage of our analysis two reviewers piloted this approach, using data extracted from the two highest-quality studies of similar interventions [55, 56]. We applied line-by-line codes, beginning with in vivo codes which closely reflected the words used in the theory reports. We then grouped and organised codes, applying axial codes reflecting higher-order themes and met to compare and contrast the resulting coding. Because this approach did not readily capture the complex and well-described interrelationships between theoretical constructs present in the reports, we instead decided to develop a novel diagrammatic approach to theory synthesis. This methodological innovation allowed us to summarise the components of each intervention’s theory of change and the explicit and/or implied causal relationships between them, drawing on text and diagrams present in the studies. Summarising these diagrammatically also facilitated comparison and synthesis of these components and relationships across included theories of change. Specifically, we drew diagrams of theories of change based on author text and diagrams, first for each intervention and then for overarching theories of change, which applied across multiple interventions. Like the approach we had initially planned, this novel method of theory synthesis was a form of qualitative synthesis, but one that aimed to describe theories of change primarily in terms of constructs, inter-connections and interactions rather than as narrative themes. Like conventional thematic analysis, it involved an initial stage of ‘in vivo’ coding of author descriptions to identify theories of change for each intervention (but expressed diagrammatically rather than as a set of narrative themes), followed by a stage of ‘axial’ coding to explore inter-connections between in vivo coding, identifying similarities and differences across interventions to develop overarching theories of change (again expressed diagrammatically).

Where more than one publication reported on the same intervention, reviewers drew on the theory of change descriptions from all relevant reports to inform the diagram. The two reviewers then met to compare the two diagrams for each intervention and reconcile discrepancies through discussion. Drawing on the strengths of each, we developed an overall diagram of each intervention’s theory of change, which included intervention components, mediators and moderators (where discussed by authors) and intended outcomes. Where author descriptions implied but did not explicitly state inter-relationships between components of the theory of change, reviewers made inferences and noted where the diagrams were in part based on such inferences.

Finding that the theory of change approaches underpinning the interventions were not patterned by targeted outcomes, we took an inductive approach, grouping diagrams of theories of change that shared important constructs. Then, using reciprocal translation (to identify and describe similar concepts occurring across theories of change underpinning different interventions), refutational synthesis (to identify contradictory or opposing concepts occurring across theories of change) and line of argument (to synthesise distinct elements occurring across theories of change that form part of a broader whole) approaches from meta-ethnography [57], each reviewer independently analysed the diagrams within each grouping. They did this by systematically examining the constructs and the relationships between constructs presented in each intervention-specific diagram and by examining whether they recurred, appeared only once or conflicted with those depicted in other intervention-specific diagrams within the grouping. Based on their analyses, each reviewer then independently drafted one synthesised diagram for each grouping of similar intervention theories of change.

We documented each stage of this process, noting where theory of change components or relationships between these components differed between individual diagrams within the grouping; the approach taken to synthesise these components (i.e. reciprocal translation, refutational synthesis, line of argument or the exclusion of a theory of change component); and the resulting decision for the synthesised theory of change diagram. The two reviewers then met to compare their respective synthesised diagrams for each grouping, reconciling discrepancies and drawing on the strengths of each to develop a single synthesised theory of change diagram for each theory of change grouping. To demonstrate this process, Additional file 2 presents the theory of change diagrams for each individual intervention in one grouping and the resulting diagram of the synthesised theory of change for that grouping. Each synthesised theory of change was given a descriptive title inductively drawing on the central approaches of the theories of change synthesised.

In this application of meta-ethnographic methods to the synthesis of theories of change, our first-order constructs were the theory of change information described in theory reports and represented in data extraction forms; our second-order constructs (analogous to in vivo codes) were the reviewers’ interpretations of these concepts, represented in the intervention-specific theory of change diagrams and our third-order constructs (analogous to axial codes) were the higher-order interpretations, represented by the diagrams of the synthesised theories of change developed for each inductive grouping.

## Results

### Screening

**Fig. 1:**
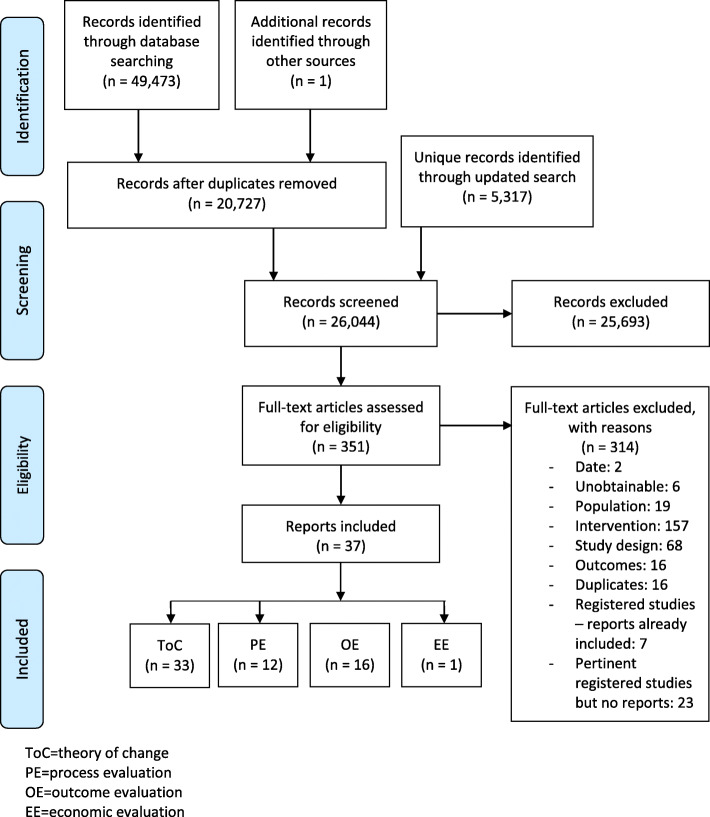
Searches and screening

Our search yielded 26,044 unique references, of which 37 were included in the overall review. Of these, 33 reported on intervention theories of change (see Fig. 1).

### Reports included in the intervention theory of change synthesis

The 33 reports included in the theory of change synthesis represent 28 unique studies and 23 interventions [55, 56, 58–87]. Two interventions—a tailored and a non-tailored version of the *Cognitive Vaccine Approach*, both reported by Davidovich et al.—share a single theory of change [70], resulting in 22 unique theories of change included in the synthesis. Table 1 summarises each intervention theory of change; evidence supporting the theory of change; how the theory of change was developed and existing scientific theories on which it draws.

**Table 1:** Summary of intervention theories of change

Intervention name	Technology	Reports	Summary of theory of change including key constructs and mechanisms	How theory of change was developed	Existing theories drawn on	Evidence supporting the theory of change
**China – Gate HIV Prevention Programme Online Intervention**	Internet	Cheng [59]	Informed by the theory of planned behaviour, the intervention targeted attitudes, subjective norms, perceived control and behavioural intentions which are posited as key determinants of health behaviours. It aimed to increase knowledge and reduce misconceptions. Part I aimed to engage participants and increase HIV risk perceptions by presenting realistic scenarios and to increase awareness of community norms by presenting peer attitudes towards behavioural decisions. Part II addressed basic HIV/AIDS knowledge and transmission; presented information about the HIV epidemic amongst MSM, aiming to further increase perceptions of consequences of CAS and to promote safer sex and addressed misconceptions about sexual behaviours.	Not stated	Theory of planned behaviour	Not stated
**Cognitive Vaccine Approach** (tailored and non-tailored versions)	Internet	Davidovich [70]	There were two versions of this online cognitive behavioural intervention promoting negotiated safety (i.e. condomless anal intercourse between steady partners who are both HIV-negative). A non-tailored version delivered all modules, and a tailored version delivered general content considered relevant for all users in addition to selected modules considered relevant to the user based on a baseline questionnaire assessing their barriers to safe sex. Each module targeted specific cognitive determinants of behaviour. Informed by the IMB model, modules addressed information, motivation and behavioural skills; the motivation component was further informed by other behaviour change theories. The intervention did not focus on promoting condom use but did provide information on condoms and recommended their use where negotiated safety was not feasible. Information modules addressed how to practice-negotiated safety, aiming to increase response efficacy (comprising knowledge of and belief in benefits of this approach for protecting against HIV). Informed by the theory of planned behaviour, motivation modules aimed to correct faulty beliefs in order to shape attitudes and informed by the health belief model, motivation modules aimed to increase users’ perceptions of HIV testing benefits as well as users’ sense of vulnerability to contracting HIV from steady partners. In turn, attitudes were theorised to increase condom use intentions and attitudes, sense of vulnerability and perceived benefits of HIV testing were theorised to increase intentions to practice negotiated safety (comprising its three components of HIV testing, reaching agreements about sex outside of the relationship and warning the partner if sexual risk outside of the relationship occurred).	Modules were guided by the IMB model and content was informed by empirical research.	IMB model, operationalising the ‘motivation’ component by drawing on components of the theory of planned behaviour and the health belief model	Content was based on past research on determinants of sexual risk behaviour in steady relationships. Authors highlighted that the IMB model has been effective in promoting HIV prevention behaviours amongst various groups, including amongst gay men.
**Gay Cruise**	Internet	Kok [73]	In this online interactive simulated cruise ship, users (MISM) selected a virtual character to guide them through the intervention using scripted, tailored dialogue. This guide introduced strategies to promote consistent condom use by making condom use an automatic behaviour. The intervention addressed knowledge (about dating, sex and safer sex) via active learning; risk perceptions via consciousness raising and feedback; skills via instruction (including video instruction), feedback and reinforcement; self-efficacy via this skill-building and via modelling, reinforcement and building on the learner’s perspective; and access to condoms via addressing where to buy condoms and offering a sample package. The intervention also aimed to influence attitudes about condoms, personal and subjective norms and anticipated regret. Intermediate outcomes included the following: making the decision to use condoms, purchasing condoms and lubricant, negotiating condom use during online chatting, expressing the wish to use condoms in the user’s chat profile, carrying enough condoms and lubricant when on a date, correctly using condoms and lubricant and using condoms consistently even in difficult circumstances.	At part of the systematic ‘intervention mapping’ process for intervention development, researchers searched the literature for behaviour change methods that could address programme objectives; drew on existing theory; and, in consultation with experts in MSM, chatting, e-dating and the Internet, selected theoretically informed strategies to achieve programme objectives.	Transtheoretical model and social cognitive theory	Not stated
**HealthMindr**	Smartphone/ mobile app	Sullivan [84] Jones [66]	Features of this mobile app included the following: risk assessments used to provide tailored prevention suggestions, with customisable assessment reminders; screeners assessing eligibility for PrEP and PEP; tailored recommendations for HIV testing frequency; identification of HIV testing options tailored to participant preferences and testing location details and map; an HIV test planner with customisable reminders; test kit, condom and lubricant ordering; substance use/mental health screening; service directory and a feature allowing users to submit questions to study staff. Based on social cognitive theory, risk assessments are theorised to lead to feedback and self-regulation, and for each of several targeted health behaviours app features were designed to promote four mechanisms of change: goal-setting, self-efficacy, outcome expectations and self-regulation. Amongst the targeted health behaviours were making an HIV testing plan, using condoms, self-screening for PrEP, and—for those living with HIV—seeking HIV care. The authors described the theory of change for the behaviour of HIV testing as an example: The ‘Make a plan’ feature promoted goal-setting; presenting information and several testing options promoted self-efficacy; information about the benefits of testing promoted positive outcome expectations; and a customizable reminder system for testing promoted self-regulation.	Not stated	Social cognitive theory	Not stated
**Hot and Safe M4M** (website name)	Internet	Carpenter [68]	Based on the IMB model, this module-based intervention aimed to reduce risk of HIV and other STIs by addressing information, motivation and behavioural skills. The information component aimed to increase knowledge of risk factors. Intervention activities assessed readiness to change and incorporated stage-based and (informed by motivational interviewing approaches) decisional balance exercises to increase motivation. Informed by motivational interviewing, the intervention also assessed HIV risk factors and targeted feedback based on user responses, and identified perceived barriers to change in order to increase self-efficacy for change. Skills training addressed skills for safer behaviour; topics addressed in communication skills training included communication about HIV status, condom use negotiation, sexual rights, differences in communication styles and sexual safety contracts.	Not stated	IMB model and motivational interviewing	Authors cited references for IMB model as an effective approach for HIV prevention.
**Internet-Based Safer Sex Intervention** (no name)	Internet	Milam [76]	This intervention aimed to reduce STI and HIV transmission by targeting the following behaviours amongst HIV-positive MSM: condom use, disclosure to sex partners, ART initiation and reduced use of drugs and alcohol. Based on their responses to monthly sexual behaviour surveys, users were directed to static web pages tailored to their risk of STI and HIV transmission. Informed by social cognitive theory and the transtheoretical model, the intervention used messaging that took into account the user’s current behaviour and intent related to the targeted behaviour change.	Not stated	Social cognitive theory and the transtheoretical model	Not stated
**Keep it Up!**	Internet	Mustanski [77] Greene [71] Mustanski [79] Mustanski [80] Madkins [64]	Participants were recruited to this online modular HIV prevention intervention following a negative HIV test, a time when they were believed to be particularly receptive to HIV prevention efforts. Informed by the IMB model, intervention activities were theorised to engender knowledge, motivation and behavioural skills and self-efficacy. In the model, self-efficacy comprised both confidence in enacting safer sex behaviours such as condom use and discussing safer sex with a sex partner, and the ability to avoid condomless anal intercourse when condoms were not available or when facing pressure from a partner. Activities involving reflection were theorised to impact behavioural intentions, an examination of safer sex practices (e.g. pros and cons of condom use), perceived social norms (amongst partners, friends and family) and a sense of vulnerability which, along with identifying sources of support, were theorised to contribute to motivation. Booster sessions were designed to reinforce learning and provide additional information on HIV prevention.	Not stated	IMB model	Not stated
**MOTIVES**	Text messaging	Linnemayr [74]	This text message-based HIV prevention intervention aimed to provide prevention information and to have participants engage with and retain it, increase HIV-testing frequency and support users in staying HIV-negative. Weekly, the user received a text message providing HIV prevention information. Informed by behavioural economics, which suggests that ‘nudges’ can be effective in changing behaviours, a follow-up text message 2 days later asked the user a question about the information received and told them that a correct answer would increase their chance of winning a prize. The ‘nudge’ of an opportunity to win a prize was theorised to incentivise ongoing engagement with the intervention, increasing knowledge retention to supporting behaviour change. Informed by behavioural economics research suggesting that prompt and frequent feedback is important for behaviour change and can help keep users engaged, users received a message immediately after sending their response that indicated whether they were correct and provided a link with more information. If they were correct, the messages also told them they had increased their chances of winning the next prize drawing. Informed by principles of behavioural economics, the intervention provided frequent prizes in order to increase salience, which the authors theorised kept the desired behaviour high on the user’s list of priorities. Users also received a text message reminder every 2.5 months to test for HIV. The intervention also aimed to increase self-efficacy as a mediator of behaviour change. The theory underpinning this intervention also accounted for variation depending on participant characteristics, identifying socio-demographics, acculturation, mental health and substance abuse as potential moderators of its impact.	Not reported	Behavioural economics	Studies suggest that lotteries can be effective in influencing a range of health behaviours, including sexual behaviour, and there are promising early results from a study aiming to improve antiretroviral adherence using this approach. Other studies suggest that behavioural economics approaches of delivering feedback promptly and frequently can support engagement and is important for behaviour change.
**myDEx**	Internet	Bauermeister [55] Bauermeister [60]	This online module-based comprehensive sex education intervention aimed to improve psychological well-being and reduce HIV risk via behaviour change (increasing condom use, increasing HIV/STI testing and reducing unprotected anal sex), increasing PrEP awareness/uptake/adherence and decreasing alcohol and drug use before sex. It was informed by the notion that decision-making is shaped by both affective and cognitive motivations, that affective motivations can be processed more quickly and therefore might drive decision-making and that when cognitive and affective motivations are less aligned, there is less of a correspondence between intentions and behaviour. The intervention therefore aimed to increase users’ cognitive motivations and to influence affective motivations. Content included information provision, activities and videos and via the latter two also aimed to build HIV risk reduction skills and promote self-reflection. Content targeting cognitive motivations focused on risk reduction attitudes (comprising attitudes towards consistent condom use, status disclosure and HIV/STI testing), risk reduction norms (comprising subjective norms, personal norms such as anticipated regret and descriptive norms, i.e. perceived prevalence of behaviours within one’s social group) and perceived behavioural control to engage in risk reduction behaviours (i.e. the ability to elicit/disclose HIV status, negotiate condom use and delay sexual intercourse). Attitudes and norms were theorised to each influence each other, and all three constructs were theorised to influence behavioural intentions. Acknowledging the influence of affective motivations on behavioural intentions, the intervention also addressed relationship ideation, anticipated regret, limerence and decisional balance to forego condoms. Behavioural intentions were theorised, in turn to directly influence HIV risk reduction behaviours. The theory underpinning this intervention also accounted for variation depending on participant characteristics: psychological risk correlates, which include sexuality-related stressors (e.g. internalised homophobia), psychological distress (e.g. depression, anxiety, loneliness and low self-esteem) and substance use and abuse were theorised to influence regulation of affective motivations and therefore behavioural control, affecting risk behaviours. Type of sexual partner (e.g. casual encounter, romantic interest or friend with benefits) was theorised to affect perceived behavioural control and the relationship between behavioural intentions and actual behaviours.	Not stated	Dual processing cognitive-emotional decision-making framework; and the IMB model	Research suggests decision-making can be affectively rather than analytically driven because affective motivations might be processed more quickly than cognitive motivations. The authors also noted that intentions correspond less with behaviour when affective and cognitive motivations conflict; and that anticipation of an emotional reaction following an unintentional behaviour is associated with less risk-taking amongst MSM.
**MyPEEPs Mobile**	Internet	Kuhns [88]	Delivered via games, scenarios and role-plays in 4 sequential modules, this app aims to reduce sexual HIV risk and promote health behaviours amongst adolescent sexual minority men. Content delivers information on HIV/STIs amongst YMSM, promotes skill-building (for condom use, emotional regulation and negotiating interpersonal and substance-related risks) and aims to raise awareness about minority stress. A goal-setting activity running throughout the intervention aims to build knowledge, self-awareness and self-efficacy by asking participants to establish and regularly reconsider their limits and the risk they are willing to accept for different types of sexual acts. The authors also state that content addresses psychosocial and contextual factors important to young people’s vulnerability to risk, including affect dysregulation (psychosocial) and family, peer and partner relationships (contextual).	Not stated	Social-personal framework, which authors say builds on social learning theory	Intervention is based on a group-based intervention that was effective in reducing sexual risk behaviour.
**Online Mindfulness-Based Cognitive Therapy** (no name)	Internet	Avellar [58]	Though the target population was not restricted to those who have experienced bullying, the rationale supporting the intervention posited that anti-LGBQ bullying could lead to internalised homophobia (also referred to as internalised homonegativity), which could cause self-stigma, undermine self-worth and cause avoidance of emotions, thoughts and situations. In this online modular intervention, sessions 1-4 focused on teaching users to identify and understand emotional and cognitive patterns causing distress and sessions 5-8 taught users how to handle these and their effect on mood, i.e. skills for awareness, moving attention to breathing, then expanding this attention to the whole body. Via practices such as increasing awareness of ingrained routines, paying attention to and accepting sensations/feelings/thoughts in each moment without judgement, developing a third-person awareness and prioritising ‘being’ over ‘doing’ or goal-attainment, and by developing an understanding of the relationship between thoughts and moods, the intervention aimed to develop skills for reducing rumination about unpleasant experiences, reducing the time that unpleasant thoughts stay in the mind, and alleviating unpleasant thoughts, feelings and emotions. Via these skills and by reducing internalised homophobia, the intervention aimed to reduce the recurrence of depression and to improve mental health.	Intervention was modelled on an existing 8-week mindfulness-based cognitive therapy protocol found to be effective for addressing symptoms of depression and anxiety.	Mindfulness-based cognitive therapy combines mindfulness and cognitive behavioural techniques to alleviate depressive symptoms	A 2012 study found that Acceptance Commitment Therapy, of which mindfulness was a key mechanism, was effective in improving outcomes including internalised homonegativity, depression, anxiety and stress amongst LGBQ participants experiencing self-stigma related to their sexual orientation. Furthermore, the Online Mindfulness-Based Cognitive Therapy intervention was modelled on an existing protocol effective for addressing symptoms of depression and anxiety.
**People Like Us**	Internet	Tan [65]	Sexual health messages incorporated into this web drama series aimed to increase HIV/STI knowledge and risk perception; provide information on HIV/STI testing and its benefits as well as resources for HIV/STI testing and other mental health services; address homophobia and sexual identity disclosure; increase self-efficacy for negotiating safer sex and promote positive attitudes, skills and self-efficacy related to safer sex. Content incorporated modelling of safer sex behaviours. The intervention aimed to impact perceived homophobia; internalised homophobia; self-concealment of sexual orientation; connectedness to the LGBT community; HIV knowledge; HIV/STI risk perceptions; consistent condom use; STI incidence and HIV/STI testing intentions, behaviours, self-efficacy and social norms.	Not stated	Not stated	Not stated
**Queer Sex Ed**	Internet	Mustanski [78]	This comprehensive sexual health curriculum for LGBT youth, delivered via online modules, was guided by the IMB model. The IMB model posits that health behaviours result from information, motivation and behavioural skills. The authors highlighted motivation as particularly important for adolescents and posited that motivation consisted of perceived vulnerability to health problems, as well as attitudes, intentions and perceived social norms. The intervention also aimed to influence sexual health behaviours by increasing self-efficacy (specified in relation to coming out and to creating and adhering to sexual agreements); a sense of connectedness to and belonging in the LGBT community; knowledge; and behavioural skills. Specific targeted outcomes mapped on to the topics of the first 4 intervention modules (NB, outcomes were not assessed for the 5th module, addressing goal-setting) and included sexual identity, sex education, healthy relationships and safer sex.	Intervention was informed by prior mixed-methods research.	IMB model	None stated
**Rainbow SPARX**	Computer (CD-ROM), with paper-based user notebook	Lucassen [75]	*Rainbow SPARX*, a computerised CBT programme designed as a computer game, introduced six core CBT skills which were theorised to support users in addressing harmful core beliefs that affect mental health. The main CBT skills covered in the intervention were the following: relax (relaxation training); do it (e.g. behavioural training); sort it (e.g. social skills training); spot it (recognising or naming cognitive distortions); solve it (problem solving) and swap it (e.g. cognitive restructuring). Content tailored to issues and experiences of sexual minority youth targeted particular challenges facing this population such as internalised homophobia and exposure to negative attitudes about same-sex attraction. Author descriptions suggested that the intervention was theorised to work via behavioural and relaxation training and via teaching users to recognise and challenge cognitive distortions. Each user could customise their avatar using any of the customisable options regardless of whether the options were traditionally female or male, with the rationale that negative repercussions often faced by this population for non-gender-conforming behaviours could contribute to internalised negative attitudes about behaviours that were natural for these young people.	The general approach of CBT was adapted to address challenges sexual minority young people face.	CBT theory	Authors cited evidence that CBT is effective in treating depression amongst adolescents.
**Role-Playing Game**	Computer download	Coulter [62]	This role-playing game aimed to improve health of bullied sexual and gender minority youth by improving help-seeking and productive coping strategies to reduce substance use, victimisation and mental health issues. The user played a customisable character who builds a team with nonplayble characters in order to defeat robots in the Holochamber Challenge. The user was tasked with helping each nonplayable character with challenges such as bullying, confidence or anger, and if successful that character joined their team. Elements of social cognitive theory, stress and coping theory and the social and emotional learning framework were embedded in the game. Pairing the player with lonely characters was theorised to increase help-seeking intentions, self-efficacy and behaviours. Active listening and helping another character overcome were theorised to increase productive coping strategies (assessed as problem solving coping) and coping flexibility (assessed as ‘evaluative coping,’ or how well the user monitors and evaluates the outcomes of coping, and ‘adaptive coping,’ or how well the user uses an alternative coping strategy to achieve a desired outcome). Collating information about bullying and external resources was theorised to increase knowledge and use of Web-based resources. The intervention also aimed to decrease non-productive coping (assessed as passive avoidant coping). Drawing on social cognitive theory, the authors suggested that self-efficacy and social skills could be developed via behavioural rehearsal, witnessing outcomes of one’s choices and feedback. Whilst not linked directly to intervention components in the authors’ narrative, these techniques were embedded in intervention design, which included supporting nonplayable characters in productive coping (rehearsal); receiving reports on the outcomes for each character based on the user’s decisions (witnessing outcomes) and receiving hints about how to better help other characters where appropriate (feedback). Loneliness, internalised gender minority stigma and internalised sexual minority stigma were also assessed, although their relationships to other outcomes were not specified.	Not stated	Social cognitive theory, stress and coping theory and the social and emotional learning framework	Not stated
**Safe Behaviour and Screening**	Smartphone/ mobile app	Chiou [61]	The app drew on the IMB model, which posits that information, behavioural motivation and skills influence HIV prevention behaviour. App content provided information which aimed to increase knowledge. Survey measures suggest the intervention also targeted motivation (comprised of attitude towards reducing risky sexual behaviour and recreational drug use, and intention to change these behaviours) and behavioural skills for HIV prevention (including partner communication, negotiating safe sex, drug and unsafe sex refusal skills and correct condom use).	Not stated	IMB model	Not stated
**Sex Positive!**	Internet	Hirshfield [72] Hirshfield [63]	Informed by social cognitive theory and social learning theory, this intervention aimed to prevent onward HIV transmission amongst MSM living with HIV. Following the character “Guy,” a gay man living with HIV, a 6-video dramatic series sought to optimise engagement by featuring stories and characters with which target users would identify. Content focused on HIV transmission, and informed by social learning theory (which posits that people learn by observing others’ attitudes and behaviours and the outcomes of their behaviours), it used modelling to demonstrate risk reduction and health behaviours including HIV disclosure, medication adherence and discussions about safer sex. Content aimed to promote critical thinking about medication adherence, viral suppression, HIV disclosure, sexual decision-making under the influence of drugs or alcohol and serodiscordant CAS. Via modelling the videos also depict cognitive dissonance and expectation failure. Authors’ description of social learning and social cognitive theories combined with the constructs assessed in user surveys suggested critical thinking was theorised to promote self-efficacy for safer sex and for HIV status disclosure to the user’s partners; promote perceived personal and partner responsibility for preventing HIV transmission; and shape outcome expectancies for condoms, anal sex and HIV disclosure. The report also suggested that modelling of self-regulation aimed to improve skills for regulating sexual compulsivity. Taken together, these mediators were theorised to influence HIV treatment adherence, mental health, substance use, sexual behaviour and interpersonal violence outcomes. Four follow-up booster videos aimed to help sustain intervention impact over time.	Not stated	Social cognitive theory and social learning theory; authors also noted that elements of both social learning and attitude change theories informed the intervention	Not stated
**Sexpulse**	Internet	Rosser [82] Wilkerson [86]	This modular HIV prevention intervention was guided by the sexual health model, which posits that people are more likely to make decisions that are sexually healthy when they themselves are sexually healthy. The intervention addressed the following aspects of the model: (1) mental and emotional health, (2) physical health, (3) intimacy, (4) relationships, (5) sexuality and (6) spirituality. Content covered other specified topics as body image and communication, amongst others, but their relationship to the sexual health model and to the intervention was not clear. Based on the authors’ description, the theory of change underpinning the intervention seemed to be that addressing aspects of broader sexual health would support safer sexual health decision-making.	Not stated	Sexual health model	Not stated
**Smartphone Self-Monitoring** (no name)	Smartphone/ mobile app	Swendeman [85]	In this smartphone-based intervention, customisable alarms prompted the user to fill in self-monitoring surveys and participants could access a Web-based visualisation tool to view their survey responses over time and by location as well as associations between variables. Daily surveys asked about alcohol, tobacco and other drug use; sexual behaviours; and medication adherence. Surveys 4 times per day asked about physical and mental health. The intervention also included event-based reporting about stressful events, and text diary entries, both of which could be done at any time. Self-monitoring was theorised to support self-management via the user’s response to feedback deriving from self-observation. Whilst authors highlighted that mechanisms of self-monitoring interventions are not well-understood, their description suggested that processes such as the user reflecting on their behaviours in comparison with particular criteria (e.g. perceived norms or personal standards) could lead to reinforcement via self-reward or self-critique, resulting in self-regulation and ultimately self-management in four domains of HIV-related health outcomes: medication adherence, mental health, substance use and sexual risk behaviours.	Not stated	Underpinned by the notion that self-monitoring can support self-management; NB, we note that self-monitoring is a core construct of social cognitive theory [89]	In studies of alcohol, tobacco and drug abuse and sexual risk reduction HIV interventions, changes amongst control groups suggest self-monitoring (via assessments) can effectively improve targeted outcomes. Evidence suggests self-monitoring is a key component of evidence-based interventions for a range of conditions, and some evidence from meta-analyses suggests self-monitoring can be particularly effective for changing and maintaining behaviours.
**SOLVE (Socially optimised learning in virtual environments)**	Computer download	Christensen [69]	In this 3-D animated game, the user took the role of a customisable avatar and made decisions which affected the narrative in simulated settings presenting risky situations and barriers to safer sex that young adult MSM typically confront on first dates or ‘hook-ups’. Via multiple theorised pathways, the intervention aimed to decrease condomless anal intercourse thereby reducing HIV risk. Informed by the notion that shame due to ‘sexual stigma’ can contribute to HIV risk behaviours, the intervention simulated shame-inducing situations; promoted conscious acknowledgement and normalisation of the user’s desires; and role-modelled positive attitudes towards one’s self as well as comfort with the user’s sexuality and desires. Guide characters and sex partners within the game were accepting of the user’s desires and also shared them. Whilst the relationship between specific aspects of the intervention and theorised mechanisms was not explicit, the authors’ description suggested these features of the intervention aimed to decrease shame by normalising the MSMs’ desires, increasing self-worth and self-acceptance and reducing isolation and feelings of inferiority. Additionally, drawing on neuroscience research suggesting that emotions play a critical role in decision-making, *SOLVE* aimed to increase self-awareness of goals, emotions and barriers to safer sex; promote recognition of the consequences of the user’s desires; interrupt affect-based decision-making and increase self-regulation. Authors’ descriptions seemed to suggest these were accomplished by challenging user choices and exploring their consequences within the simulated scenarios. Other components of the intervention aimed to increase HIV knowledge and hone HIV risk-reduction skills and strategies.	Not stated	Theory of planned behaviour, social cognitive theory and neuroscience research suggesting that emotions play a critical role in decision-making	Two prior RCTs of similar interventions were effective in reducing unprotected anal intercourse.
**TXT-Auto**	Text messaging	Reback [81]	*TXT-Auto* aimed to reduce substance use and HIV risk amongst out-of-treatment methamphetamine-using MSM. Users received 5 automated scripted text messages per day, which included both general messages and messages tailored to the user’s risk profile. Risk profile was determined based on responses to a baseline survey assessing risks in relation to HIV status, ART adherence, drug use and sexual behaviours. Text message content was based on social support theory, social cognitive theory and the health belief model, which the authors described as complementary theories, though the constructs drawn from each theory and the intended mechanisms of change were not described. Text messages aimed to increase knowledge, and an example provided of messaging informed by social cognitive theory suggested they might also aim to increase self-efficacy. A brief weekly text-based assessment asking about methamphetamine use and HIV sexual behaviours in the past 7 days aimed to increase self-monitoring. Taken together, intervention activities aimed to decrease methamphetamine use, sex during methamphetamine use and CAS.	The theoretical constructs underpinning the intervention were selected during a pilot study, informed by evidence-based behavioural change theories with complementary designs.	Text messages were based on social support theory, social cognitive theory and the health belief model	Authors note that theoretical principles on which each behavioural change theory rests have been proven effective in multiple studies.
**WRAPP**	Internet	Bowen [67] Bowen [56] Williams [87] Schonnesson [83]	*WRAPP* was informed by social cognitive theory and the IMB model, and each of its three modules corresponded to one aspect of the IMB model. The ‘knowledge’ module was designed as the ‘information’ component and primarily addressed living with HIV and HIV prevention, aiming to increase HIV knowledge. The ‘partner’ module aimed to increase motivation (comprising outcome expectancies for risk reduction and willingness to reduce HIV risk behaviours). It addressed risk with both new and casual partners, supporting participants in clarifying long-term life goals and in considering whether these were consistent with unsafe sex. The ‘contexts of risk’ module targeted behavioural skills, supporting the user in adopting risk reduction behaviours with sexual partners met online or in a bar. Knowledge, motivation and behavioural skills were theorised to increase sexual self-efficacy (comprising mechanical self-efficacy—such as self-efficacy for correct condom use—and self-efficacy to refuse CAS), which was theorised to be a direct precursor of behaviour change.	Not stated	Social cognitive theory and the IMB model	Evidence was not discussed directly, but in a later iteration [56] the authors noted that their work extended an earlier iteration that improved HIV-related knowledge, condom use outcome expectancies and condom use self-efficacy.

*AIDS *acquired immunodeficiency syndrome*, ART* antiretroviral therapy, *CAS* condomless anal sex, *CBT* cognitive behavioural therapy, *HIV* human immunodeficiency virus, *IMB model* information-motivation-behavioural skills model,* LGBQ* lesbian, gay, bisexual and queer, *LGBT* lesbian, gay, bisexual and transgender, *MISM* men who use the internet to seek sex with men, *MSM* men who have sex with men, *PEP* postexposure prophylaxis, *PrEP* pre-exposure prophylaxis,* RCT* randomised controlled trial, *STI* sexually transmitted infection, *YMSM* young men who have sex with men

### Quality assessment

Agreement between independent reviewer assessments was good, ranging from agreement on none of the five quality criteria (*N* = 1) to 5/5 (*N* = 15), with agreement of 4/5 or above for more than three quarters of reports (*N* = 26; 79%). All disagreements on independent quality assessments were resolved by discussion. Table 2 shows quality assessment results for each theory report.

**Table 2:** Quality assessment of reports on intervention theories of change

Intervention name	Clear pathways from intervention components to outcomes	Constructs or concepts clearly defined	Clearly describes how constructs are inter-related	Clearly explains mechanisms underlying inter-relationships between constructs	Engages with how mechanisms and outcomes might vary by context	Initial agreement between reviewers (%)
**China – Gate HIV Prevention Programme Online Intervention** (no name) Cheng [59]	Y	Y	N	N	N	100
**Cognitive Vaccine Approach** Davidovich [70]	Y	Y	Y	Y	Y	100
**Gay Cruise** Kok [73]	Y	Y	N	N	N	20
**HealthMindr** Sullivan [84]	N	N	N	N	N	80
Jones [66]	Y	Y	Y	N	N	80
**Hot and Safe M4M** (website name) Carpenter [68]	Y	N	Y	N	N	60
**Internet-Based Safer Sex Intervention** (no name) Milam [76]	N	N	N	N	N	100
**Keep it Up!** Mustanski [77]	N	Y	N	N	N	80
Greene [71]	Y	Y	N	N	N	80
Mustanski [79]	N	N	N	N	N	80
Mustanski [80]	N	N	N	N	N	100
Madkins [64]	N	Y	N	N	N	80
**myDEx** Bauermeister [55]	N	Y	Y	Y	Y	100
Bauermeister [2019]	Y	Y	Y	N	Y	100
**MOTIVES** Linnemayr [74]	Y	N	N	N	Y	60
**MyPEEPS Mobile** Kuhns [88]	N	Y	N	N	N	80
**Online Mindfulness-Based Cognitive Therapy** (no name) Avellar [58]	Y	Y	N	N	N	60
**People Like Us** Tan [65]	N	Y	N	N	N	100
**Queer Sex Ed** Mustanski [78]	N	Y	Y	N	N	60
**Rainbow SPARX** Lucassen [75]	Y	Y	N	N	N	100
**Role-Playing Game** Coulter [62]	Y	Y	Y	N	N	80
**Safe Behaviour and Screening** Chiou [61]	N	Y	N	N	N	100
**Sex Positive!** Hirshfield [72]	Y	Y	N	N	N	80
Hirshfield [63]	N	N	N	N	N	100
**Sexpulse** Rosser [82]	N	N	N	N	N	80
Wilkerson [86]	N	N	N	N	N	0
**Smartphone Self-Monitoring** (no name) Swendeman [85]	Y	Y	Y	Y	N	100
**SOLVE** Christensen [69]	Y	Y	Y	Y	N	100
**TXT-Auto** Reback [81]	N	N	N	N	N	100
**WRAPP** Bowen [67] (internet-delivered risk reduction; no name; preliminary work to WRAPP)	N	Y	Y	N	N	60
Bowen [56]	Y	Y	Y	N	N	100
Williams [87] (Hope Project; extends WRAPP)	N	Y	N	N	N	80
Schonnesson [83] (SMART; Swedish adaptation of WRAPP)	Y	Y	Y	N	N	100

Quality varied notably across theory reports and clustered towards low and medium quality, with 14 reports (42%) meeting none or one of the assessed criteria [61, 63–65, 76, 77, 79–82, 84, 86–88]; 14 (42%) meeting two or three criteria [56, 58, 59, 62, 66–68, 71–75, 78, 83] and only five (15%) meeting four or five criteria [55, 60, 69, 70, 85].

Quality varied across the five criteria assessed. Nearly three quarters of theory reports (*N* = 23; 70%) clearly defined constructs or concepts and about half described a pathway from intervention components to intended outcomes (*N* = 16; 48%). Slightly more than a third clearly described how theoretical constructs were inter-related (*N* = 12; 36%). Only four (12%) described underlying mechanisms and the same number described how mechanisms and outcomes might vary by context.

### Scientific theories informing intervention design

Authors cited a number of existing scientific theories informing intervention theories of change. Interventions often drew on more than one existing scientific theory. The information-motivation-behavioural skills (IMB) model and social cognitive theory were the most commonly cited, with the former informing seven interventions [55, 56, 60, 61, 64, 68, 70, 77–80, 83, 87] and the latter informing eight [62, 63, 66, 67, 69, 72, 73, 76, 81, 84]. The former was initially developed to inform HIV prevention but has since been advanced as a model for conceptualising individual and social determinants of health behaviours more broadly [90], and the latter incorporates individual, social and structural factors shaping, and techniques for changing, health behaviours [91]. One report citing social cognitive theory also cited its predecessor [92] social learning theory [72], and one report cited the social-personal framework which the authors describe as building on social learning theory [88]. The *Smartphone Self-Monitoring* intervention was underpinned by the theorised role of self-monitoring in supporting self-management [85], and we noted that self-monitoring is a core construct of social cognitive theory [89].

Other scientific theories of behaviour commonly used to inform health interventions informed between one and three interventions each: the health-belief model [70, 81], which focuses on the role of individual beliefs about health problems [93]; the theory of planned behaviour [59, 69, 70], which takes into account individual and social factors and theorises that intentions and perceptions of behavioural control are the direct precursors to behaviours [94] and social support theory [81], which theorises that support or perceptions of support from people who are trusted can reduce the stress of, and improve coping with, difficult events [95]. Content for the *Sexpulse* intervention was informed by the sexual health model, which identifies ten components essential to healthy sexuality [96], theorising that ‘sexually healthy persons are more likely to make sexually healthy decisions’ ([82], p. 2). In addition to social cognitive theory, the *Role-Playing Game* intervention was informed by stress and coping theory and by the emotional learning framework [62]. In regard to bullied youth, the former posits that youths’ appraisals of their experience predict their coping strategies, with those who blame themselves, perceive little control and view bullying as a threat instead of a challenge tend to use non-productive coping strategies. The emotional learning framework specifies four health-promoting competencies, described as ‘awareness of self and others, responsible decision making, positive attitudes and values, and social interaction skills’ ([62], p. 6).

The *MOTIVES* intervention was rooted in behavioural economics [74], which examines how actors make decisions other than via conscious reasoning. Two interventions were underpinned by theory on interactions between cognitive and emotional factors: *myDEx* was informed by a ‘dual processing, cognitive-emotional decision making framework’ (p. 4), which recognises that affective states (emotions) and cognitive states (thinking) both influence decision-making, and that the former might be processed more quickly than the latter [55]. The *SOLVE* intervention was informed by learning from neuroscience highlighting the important role emotions play in decision-making [69].

Scientific theories of behaviour change were also cited, such as the transtheoretical model [73, 76], which maps stages and processes by which people change their behaviours [97]. The *Hot and Safe M4M* intervention incorporated strategies from motivational interviewing by assessing, and delivering exercises based on, participants’ ‘readiness to change’ ([68], p. 552). Two interventions were rooted in cognitive behavioural therapy (CBT), which examines inter-relationships between cognitions and behaviours and how these may be modified: the *Online Mindfulness-Based Cognitive Therapy* integrated mindfulness and CBT approaches [58] and the *Rainbow SPARX* intervention used a computer game format to deliver CBT [75].

As evidence to support intervention components and theories of change, theory reports often cited evaluations of earlier iterations of the current intervention or of similar interventions, or previous research on the scientific theories in question. Where authors discussed how the intervention theory of change was developed, these were commonly informed by formative research, existing interventions, literature on the needs of the target group, components of the scientific theories underpinning the intervention or a combination of these.

### Theory of change synthesis

All intervention theories of change identified intended intervention components, mediators and outcomes which could be incorporated into an intervention-specific theory of change diagram except for the theory underpinning the *Internet-Based Safer Sex Intervention* [76], which did not identify mediators. Two theories of change also identified participant characteristics theorised to moderate the relationship between the intervention and its intended outcomes [55, 60, 74].

Our grouping of theories of change based inductively on their key constructs resulted in three groups of intervention theories of change. The largest, the ‘cognitive/skills’ theory of change grouping [55, 56, 59, 61, 65, 67, 68, 70–73, 77–80, 83, 84, 87, 88], was informed largely by social cognitive theory and the IMB model. The second grouping drew on two theories of change driven primarily by self-monitoring (the ‘self-monitoring’ theory of change grouping) [81, 85], and the third drew on two theories of change based on cognitive therapy approaches (the ‘cognitive therapy’ theory of change grouping) [58, 98]. Five theories of change did not fall within any of the three inductive groupings [62, 69, 74, 76, 82, 86]. The ‘cognitive therapy’ theory of change grouping comprised only mental health interventions. The other two theories of change groupings were not patterned by intended outcomes.

### ‘Cognitive/skills’ synthesised theory of change

**Fig. 2:**
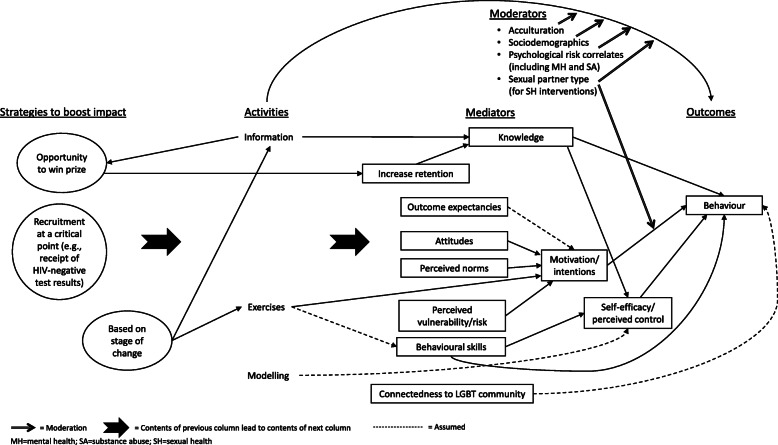
‘Cognitive/skills’ synthesised theory of change

We drew on thirteen intervention theories of change, which varied in quality from low to high, to develop a diagram for the ‘cognitive/skills’ grouping (Fig. 2) [55, 56, 59–61, 63–68, 70–73, 77–79, 83, 84, 87, 88]. Ten of the 13 theories of change in this grouping referenced social cognitive theory [56, 63, 66, 67, 72, 73, 83, 84, 87] and/or the IMB model [55, 56, 60, 61, 64, 67, 68, 70, 71, 77–80, 83, 87], often in combination with other scientific theories. Three referenced the theory of planned behaviour [59, 70, 88] and one referenced no existing scientific theories but shared key constructs with other theories of change in this grouping [65]. Whilst other interventions also drew on these scientific theories, the theories of change in this grouping shared as core components constructs that are key to these three scientific theories. All interventions represented in this grouping targeted sexual health outcomes, either alone or in combination with substance use or both substance use and mental health. Two interventions not included in this grouping were primarily rooted in different approaches or scientific theories but included components both overlapping with and complementing those in this grouping [74, 76]. Using the line-of-argument approach, we drew on these to augment findings within the ‘cognitive/skills’ grouping and these additions (theorised moderators and the provision of information based on assessed stage-of-change) appear in the overall diagram.

Interventions in this grouping provided information and exercises and incorporated modelling techniques. These activities were theorised to impact on mediators including knowledge, outcome expectancies, attitudes (including internalised homophobia [65, 78]), perceived norms, perceived vulnerability/risk (a construct combining via reciprocal translation those of perceived vulnerability [64, 70, 71, 77–80] and risk perceptions [59, 65, 73]) and behavioural skills. Two interventions whose theories of change informed this grouping aimed to impact connectedness to the LGBT community [65, 78], portrayed as a mediator in the synthesised diagram though the reports were not explicit about how intervention activities aimed to engender this connectedness or how it might be related to sexual health outcomes.

We inferred that exercises would develop behavioural skills, indicating this inference by use of a dotted line in Fig. 2, though behavioural skills could be developed through other activities. For example, in the video-based *Sex Positive!* intervention characters modelled behaviours and this was theorised to increase self-regulatory skills [72]. Theory reports did not describe clear and recurring pathways from modelling to mechanisms of change, but we inferred (denoted by use of a dotted line) from recurring descriptions that these aimed to promote self-efficacy [72, 73].

Theory reports differed as to the activities intended to influence outcome expectancies, attitudes, perceived norms and perceived vulnerability/risk. We portray the general relationship between the intervention and these mediators in the diagram by use of a block arrow. Knowledge and behavioural skills were both described as impacting behaviour either directly or via self-efficacy, so both pathways are depicted in the synthesised diagram.

Outcome expectancies—positive and negative expectations of the results of a particular behaviour [91]—were theorised as a component of motivation in the theory of change for the *WRAPP* intervention [56] and this was also implied in the report for the *Sex Positive!* intervention [72]. The theory of change for *HealthMinder* highlighted outcome expectations [84] as a key mediator without discussing motivation. Given these differences, we included outcome expectancies as a mediator and made the assumption that it influences motivation in the synthesised diagram.

Motivation and self-efficacy both featured prominently in this grouping’s intervention theories of change. Whilst some reports suggested that motivation and self-efficacy influence each other in one direction or the other [56, 68], most did not address their inter-relationship, either portraying the constructs as independent [78, 79] or including only one [70, 72, 73, 84]. The synthesised theory of change diagram therefore portrays both constructs as impacting behaviour independently via a line-of-argument synthesis.

Whilst some intervention theories of change included either motivation [59, 65] or intentions [56, 67, 68, 83, 87], those that included both variably portrayed these constructs as influencing each other in one direction or the other [55, 60, 78] or both combined into one construct [61, 70]. We have therefore combined these constructs via reciprocal translation in the synthesised diagram.

We interpreted the construct of perceived behavioural control [55, 70] to be similar to the concept of self-efficacy because the latter was variously described as including an ‘ability to refuse to have anal sex if a condom was unavailable’ ([56], p. 7), ‘confidence in practicing safer sex behaviours’ ([77], p. 3003) and/or the ‘ability to avoid the situational temptation to have unprotected sex’ ([77], p. 3003). Self-efficacy and perceived control were therefore merged via reciprocal translation in the synthesised theory of change diagram.

Theorised moderators are shown at the top right of Fig. 2. In a few cases, theory reports discuss how these might operate [55, 74]. Bauermeister et al. theorise that MSM’s ability to enact sexual risk reduction behaviours might vary by partner type (casual encounter, romantic interest or friend with benefits) and that MSM experiencing stressors related to being a sexual minority, experiencing psychological distress or using alcohol or drugs might have less behavioural control, limiting their ability to ‘regulate their affective motivations’ (p. 5) and enact sexual risk reduction behaviours [55]. Linnemayr et al. theorise that participants in the MOTIVES intervention (which is not included in this grouping but augments its findings via line-of-argument synthesis) who are more acculturated to the USA and can more easily communicate with healthcare providers might face fewer barriers to HIV testing, thus benefiting less from the intervention [74].

Finally, some interventions in this grouping [68, 77] and some from outside of it [74, 76] aimed to boost intervention impact, for example, by recruiting participants at a critical point for behaviour change [77]; delivering content based on the participant’s assessed stage-of-change [68, 76]; or using prize lotteries to incentivise knowledge retention [74].

### ‘Self-monitoring’ synthesised theory of change

**Fig. 3:**
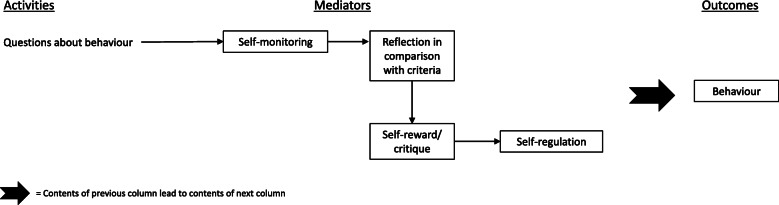
‘Self-monitoring’ synthesised theory of change

We defined a grouping focused on theorised ‘self-monitoring’ (Fig. 3) by synthesising theories of change underpinning *Smartphone Self-Monitoring* [85], an intervention addressing all three health outcomes examined in our review, with a theory report assessed as of high quality, and the *TXT-Auto* intervention [81], a sexual health and substance use intervention with content tailored by the user’s risk profile, with a theory of change description assessed as of medium quality. The synthesised theory of change posits that self-monitoring via frequent behavioural surveys prompts the user to reflect on their behaviour and reinforces the desired behaviour, which increases behavioural regulation and leads to behaviour change.

Both interventions in this grouping asked the user questions about their behaviour to prompt self-monitoring, and both were driven by self-monitoring as a key mechanism. Frequency of self-monitoring varied by study and outcome, ranging from four times per day [85] to weekly [81]. The theory report for *Smartphone Self-Monitoring* suggested the need for users to first establish criteria for their desired behaviour, such as personal norms or standards, against which they can assess their actual behaviour [85]. However, there was no evidence that this was a separate stage of the intervention so this stage does not appear in the synthesised diagram. The synthesised theory of change posits that behavioural questions result in self-monitoring, which prompts reflection in terms of pre-established criteria. This reflection is theorised to result in either self-reward or self-critique, generating enhanced self-regulation, a theoretical mediator of behaviour change.

Whilst both theory reports included in this grouping suggest processes of change are more complex than depicted in this synthesised theory, neither specified further self-monitoring mechanisms prospectively. The theory of change underpinning *TXT-Auto* suggested the intervention worked via two non-intersecting pathways, one captured in the ‘social/skills’ synthesised theory of change and one featuring self-monitoring. Arguing that pathways from self-monitoring to behaviour change are underdeveloped, Swendeman et al.’s research drew on qualitative data to further explicate the theory of change for *Smartphone Self-Monitoring* [85].

### ‘Cognitive therapy’ synthesised theory of change

**Fig. 4:**
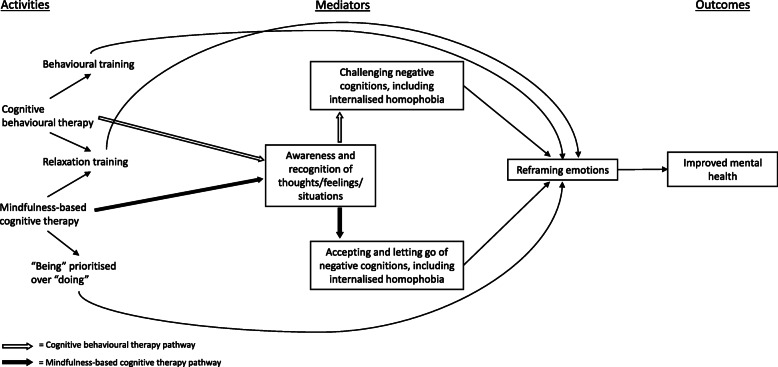
‘Cognitive therapy’ synthesised theory of change

We drew on theories of change underpinning *Online Mindfulness-Based Cognitive Therapy* [58] and *Rainbow SPARX* [75], a computer game intervention, to develop a ‘cognitive therapy’ grouping (Fig. 4). Descriptions of both were assessed as of medium quality. These interventions were the only two in this review that targeted mental health outcomes alone, and both drew on cognitive therapy techniques to reduce depression and improve mental health.

Activities in the synthesised theory of change diagram include CBT approaches (forming the core of the *Rainbow SPARX* intervention) [75] and mindfulness-based cognitive therapy, which combines mindfulness with cognitive behavioural techniques [58]. Whilst we do not detail all activities comprising each of these approaches in the synthesised theory of change diagram, we include key components of each: behavioural training (CBT), an emphasis on prioritising ‘being’ over ‘doing’ (mindfulness-based cognitive therapy) and relaxation training (both approaches).

Theory of change descriptions for both interventions referred to recognising, paying attention to or developing awareness of thoughts, feelings and situations, with *Rainbow SPARX* [75] then challenging negative cognitions and *Online Mindfulness-Based Cognitive Therapy* focusing on accepting and ‘letting go’ of them [58]. We use differently shaded block arrows in Fig. 4 to delineate these distinct pathways. Both sought to reduce internalised homophobia and mitigate its effects on health [58, 75]. The report for *Rainbow SPARX* suggested this was done by providing information to promote young people spending more time with those who accepted them and reducing exposure to homophobic bullying [75], whilst the report for *Online Mindfulness-Based Cognitive Therapy* did not make clear how this would be achieved [58]. Descriptions of both interventions’ theories of change suggest that ultimately the reframing of distressing emotions is theorised as a key mechanism for improving mental health.

### Intervention theories of change not included in synthesised models

Whilst informed by the sexual health model, the theory of change underpinning the *Sexpulse* intervention did not specify components or mechanisms and so could not be synthesised [82, 86]. Four other interventions, with theory reports ranging from low to high quality, did not fit within any of the three inductive theory of change groupings [62, 69, 74, 76]. These interventions cut across the targeted health outcomes. Whilst some components of their theories of change overlapped with, or complemented, the above groupings, theories of change for these interventions featured elements that differentiated them from those included in the groupings. The *MOTIVES* [74] intervention was based on behavioural economics, and the *SOLVE* intervention [69] was driven by the theorised role of emotions in decision-making, featuring ‘shame associated with sexual stigma’ (p. 3) as a key mediator. Drawing on social cognitive theory, stress and coping theory and the social and emotional learning framework, the *Role-Playing Game* aimed to improve mental health and reduce substance use via use of productive coping strategies with a focus on help-seeking [62]. Informed by the transtheoretical model, the theory report for the *Internet-Based Safer Sex Intervention* [76] suggested that the intervention was primarily based on a stages-of-change approach, tailoring content based on responses to monthly sexual behaviour surveys. The *MOTIVES* intervention [74] and *Internet-Based Safer Sex Intervention* [76] theory reports augmented the ‘cognitive/skills’ synthesised theory of change diagram.

## Discussion

### Summary of findings

For this review, we synthesised the theories of change underpinning e-health interventions targeting HIV/STIs, sexual risk behaviour, substance use and mental ill health amongst MSM. We developed a novel approach to do so by using diagrams rather than narrative themes to summarise theories of change within and across interventions.

Whilst we have previously conducted reviews synthesising intervention theories of change using line-by-line coding of descriptive text [51, 52], we found that this approach did not capture the often precisely described and complex inter-relationships between theoretical constructs presented in the body of literature for this review. Theories of change included in our past reviews, which addressed the integration of health and academic education [52] and positive youth development interventions [51], were relatively simple and either not significantly informed by existing scientific theories [52] or informed by theories that are not typically portrayed visually [51]. In contrast, theories of change underpinning the e-health interventions included in this review were more complex, more explicitly theorised and largely drew on existing scientific theories which are typically conceptualised in terms of diagrams indicating relationships between their theoretical constructs, with constructs widely recognised and understood and therefore not always discussed at length. Whilst thematic analysis is a good way of rendering explicit what is implicit, it is less appropriate where the literature itself is more explicit. We therefore developed a novel method of theory of change synthesis in which we created diagrammatic summative logic models of intervention theories of change. By inductively grouping these models according to their core constructs and using meta-ethnographic approaches, we identified three emergent theoretical approaches underpinning the included interventions and we created synthesised models of each approach. We have thus synthesised theories of change underlying interventions with similar approaches. These summarise and integrate existing theories of change rather than providing a novel overarching theory of change for such interventions.

Social cognitive theory and the IMB model featured most prominently in intervention theories of change and informed a small majority. Whilst reports cited a number of other existing scientific theories, each informed only a few interventions at most. In the ‘cognitive/skills’ synthesised grouping, based on theory of change descriptions assessed as ranging from low to high quality, information and exercises are theorised to influence behavioural skills and a range of distal cognitive mediators theorised to influence behaviour via motivation/intention and self-efficacy/perceived control. The synthesised theory of change suggests that particular strategies can boost intervention impact, whilst other factors relating to the participant or (for sexual health interventions) their partner moderate impact.

Though represented in fewer interventions, two other distinct theories of change groupings emerged. The ‘self-monitoring’ grouping, based on theory of change descriptions assessed as of low and high quality, focuses more narrowly on the role of self-monitoring in triggering reflection, self-reward/critique and behavioural self-regulation. Theory reports suggest this synthesised diagram represents the basic components of more complex cognitive pathways [85] and can sit alongside other distinct mechanisms of behaviour change in intervention theories of change [81]. Underpinning interventions focused solely on mental health and based on theory of change descriptions assessed as of medium quality, the ‘cognitive therapy’ grouping is rooted in cognitive therapy techniques which can be augmented by mindfulness. In this approach, activities promote awareness and recognition of the participant’s thoughts, feelings and situations and, via either challenging or accepting negative cognitions, aim to reframe negative emotions to improve mental health.

Theories of change included in this review were informed by scientific theories that have been associated with greater impacts in reviews of e-health interventions specifically, including the transtheoretical model [41, 45] and the theory of planned behaviour [45], though these approaches underpinned a minority of interventions. Several also featured modelling and self-monitoring, behaviour change techniques [99] which some evidence suggests might be effective in e-health interventions [45, 100].

As is common with e-health behavioural interventions [45, 48], the theories of change underpinning interventions in this review tended to rely on individually focused psychological theories of behaviour or behaviour change. Whilst these are unlikely to address structural factors contributing to the syndemic such as marginalisation, homophobia and discrimination [19, 101], the accessibility and anonymity of e-health interventions offer one approach to reducing barriers to service access stemming from stigma and discrimination [26] and might form an important element of a broader mix of interventions addressing individual and structural factors.

Furthermore, we found that included interventions drawing on multiple theoretical approaches [55, 58, 65, 69, 75, 78] took into account sexual minority-related stressors. One accounted for such stressors as a theorised moderator of intervention impact [55], whilst others aimed to increase connectedness to the LGBT community [65, 78] and reduce internalised homophobia [58, 65, 75, 78] and associated shame [69] to reduce sexual risk [65, 69, 78] and improve mental health [58, 75]. However, theory reports included little discussion of the relationships between these constructs, limiting our ability to explicate their roles in the synthesised theories of change.

It was notable that some existing scientific theories that informed theories of change were theories of behaviour change (for example, CBT theory) whilst others were theories of behaviour and its determinants (for example, the health belief model). Authors generally did not report drawing on scientific theories oriented more explicitly towards selecting strategies for enacting behaviour change, such as the Behaviour Change Wheel [102] or the Elaboration Likelihood Model [103]. Intervention developers might usefully draw on such models.

Our synthesis aimed to develop overarching theories of change for different sub-types of e-health interventions to address HIV/STIs and sexual risk, substance use and mental ill health amongst MSM, which we hope will help inform future interventions. Intervention developers might also draw on existing scientific theories which aim to integrate existing scientific theories of behaviour and behaviour change, such as the PRIME and COM-B models [102, 104].

### Limitations

Our synthesis is limited by the quality of the existing theory reports, which did not often describe clear pathways from activities to intended outcomes. In some cases, reviewers inferred relationships between theory of change constructs (denoting assumptions in the theory of change diagrams), and in others the relationships between specific activities, mediators and outcomes could not be determined. Whilst our approach to theory of change synthesis enabled us to systematically explore constructs and the relationships between them across intervention theories of change, synthesised diagrams do not capture aspects that theory reports suggest influenced theories of change where their role was not clear enough to be included in intervention-specific diagrams.

We did not assess the parsimony of theories of change, because we have found in past reviews that this is very difficult to consistently operationalise as a criterion of quality assessment; however, this is an important feature of theories of change. We also did not aim to systematically assess the evidence base for each of the scientific theories underpinning the intervention theories of change, because this was outside the scope of feasibility for this review and would require assessing not only the evidence for the scientific theory but also the evidence for the application of that theory to the outcomes targeted in the theories of change it underpins.

## Conclusions

e-Health interventions are a promising approach for reaching MSM with targeted health interventions [26], and existing evidence drawn from general or other populations suggests they might be effective in reducing sexual risk behaviour [39, 41] and substance use [30] and addressing common causes of mental ill health [31–37]. Our synthesis has identified three distinct theory of change pathways underpinning existing e-health interventions for MSM targeting sexual health, substance use and mental health outcomes, two of which underpin interventions targeting all three of these outcomes. The synthesised theories of change could provide a framework for the development of theory-driven e-health interventions holistically addressing these multiple, often co-occurring health issues amongst MSM, which can be augmented by familiarisation with the scientific theories on which they are based to inform a nuanced understanding of these theoretical underpinnings and how they can be most usefully applied in specific interventions. In the case of the ‘cognitive/skills’ synthesised theory of change, this would likely involve selecting a subset of mediators on which to focus, because a single intervention would not be expected to address the full range of constructs presented in this synthesised theory of change. However, our broader systematic review should first synthesise evidence on intervention effects to assess the potential of such an intervention.

Our findings suggest that the quality of existing theory reports is low- to medium, with limited discussion of the inter-relationships between theoretical constructs and little attention to how mechanisms might vary by context. Improving the quality of theory reports would enable a better understanding of how interventions are intended to work and the evidence supporting this. It would also facilitate evaluations which can go beyond outcome assessment to identify which components are triggering which mechanisms of change, and to what effect [105]. In particular, we recommend that intervention developers provide clear theories of change for their interventions, informed by existing scientific theories of behaviour and behaviour change relevant to the approach of the intervention and to the outcomes it seeks to address. Such theories of change can ensure intervention activities align with their intended outcomes, and can ensure that evaluations are focused on the most appropriate measures of implementation, mediators and outcomes and consider how mechanisms might vary by context and/or population.

The novel method of theory of change synthesis developed for this review worked well for the theories of change underpinning included interventions. These were largely driven by existing scientific theories and featured constructs commonly underpinning behaviour change interventions such as self-efficacy, motivation, behavioural intentions, attitudes and perceived norms. This approach can be used for similar reviews in which line-by-line coding does not effectively capture the relationships between theoretical constructs. Our forthcoming synthesis of outcome evaluations of e-health interventions addressing sexual health, substance use and/or mental health amongst MSM will aim to explore whether particular theoretical approaches are associated with greater impact.

## Supplementary Information

**Additional file 1.** Search terms and strategy for Medline database.

**Additional file 2.** Individual and overarching theory of change logic models for the ‘Self-monitoring’ theory group.

## Data Availability

Full details of our search strategy are available at the London School of Hygiene & Tropical Medicine’s Data Repository [50].

## Abbreviations

AIDS: Acquired immunodeficiency syndrome
CBT: Cognitive behavioural therapy
HIV: Human immunodeficiency virus
IMB model: Information-motivation-behavioural skills model
MSM: Men who have sex with men
STI: Sexually transmitted infection
