# Supplementary material for: Theories of change for e-health interventions targeting HIV/STIs and sexual risk, substance use and mental ill health amongst men who have sex with men: systematic review and synthesis
Source: Syst Rev. 2021 Jan 11;10:21. doi: 10.1186/s13643-020-01523-2 (PMC7798186; doi:10.1186/s13643-020-01523-2)
Supplement: Supplementary file 1 — Additional file 1. Search terms and strategy for Medline database. [file 13643_2020_1523_MOESM1_ESM.docx]

# Additional file 1. Search terms and strategy for Medline database

This additional file provides full details of the search string used for the search of the Medline database, with the date of the original search and the number of references (in parentheses at the end of each row) returned. The search of this database was subsequently updated on 22 April 2020 with coverage dates of 1946 to 21 April 2020.

| Database name | Medline |
| --- | --- |
| Database platform | OvidSP |
| Dates of database coverage | Ovid MEDLINE(R) and Epub Ahead of Print, In-Process & Other Non-Indexed Citations and Daily 1946 to October 22, 2018 |
| Date searched | 23/10/2018 |
| Searched by | JF |
| Number of results | 4701 |
| EndNote import order | 1 |
| Number of results once duplicates removed | 4596 |
| Search strategy notes | Search lines ending in a ‘/’ are subject heading searches. Search lines beginning ‘exp’ are exploded subject heading searches. Search lines ending in .ti,ab. search in the title and abstract only. or/*x-y* combines search sets in the range *x-y* with Boolean operator OR. * is used for truncation of words. # is used for a compulsory wildcard. ? is used for an optional wildcard. |

- - 1. Homosexuality/ (12169)
    2. Homosexuality, Male/ (13445)
    3. exp "Sexual and Gender Minorities"/ (3131)
    4. Bisexuality/ (3695)
    5. Transsexualism/ (3421)
    6. gender identity/ (17248)
    7. Health Services for Transgender Persons/ (92)
    8. exp Sex Reassignment Procedures/ (550)
    9. homosexual*.ti,ab. (13006)
    10. gay.ti,ab. (9392)
    11. "men who have sex with men".ti,ab. (9288)
    12. MSM.ti,ab. (8276)
    13. bisexual*.ti,ab. (7793)
    14. gbMSM.ti,ab. (42)
    15. (transgender* or trans-gender*).ti,ab. (3923)
    16. (transsexual* or trans-sexual*).ti,ab. (2333)
    17. (transm#n or trans-men or trans-man).ti,ab. (209)
    18. (transwom#n or trans-wom#n).ti,ab. (220)
    19. (transfemale? or trans female?).ti,ab. (19)
    20. trans people.ti,ab. (82)
    21. trans person.ti,ab. (3)
    22. tgm.ti,ab. (334)
    23. tgw.ti,ab. (180)
    24. gender identity.ti,ab. (2272)
    25. cross gender.ti,ab. (256)
    26. sex reassignment.ti,ab. (516)
    27. gender reassignment.ti,ab. (270)
    28. gender dysphoria.ti,ab. (646)
    29. gender transition.ti,ab. (89)
    30. queer.ti,ab. (905)
    31. sexual-minorit*.ti,ab. (1751)
    32. gender-minorit*.ti,ab. (304)
    33. LGBT*.ti,ab. (1350)
    34. or/1-33 [MSM] (62357)
    35. exp telemedicine/ (23614)
    36. ccbt.ti,ab. (144)
    37. (ehealth or e-health or electronic health*).ti,ab. (15158)
    38. (etherap* or e-therap* or electronic therap*).ti,ab. (426)
    39. (eportal or e-portal or electronic portal).ti,ab. (1012)
    40. telehealth*.ti,ab. (3111)
    41. telemed*.ti,ab. (9034)
    42. telemonitor*.ti,ab. (1239)
    43. telepsych*.ti,ab. (514)
    44. teletherap*.ti,ab. (1309)
    45. icbt.ti,ab. (539)
    46. (mhealth or m-health).ti,ab. (2109)
    47. or/35-46 [GENERAL E-HEALTH] (45055)
    48. cell phone/ (7494)
    49. wireless technology/ (2864)
    50. exp microcomputers/ (19620)
    51. cellphone.ti,ab. (178)
    52. computer*.ti,ab. (277170)
    53. (ipad or i-pad).ti,ab. (1036)
    54. (iphone or i-phone).ti,ab. (634)
    55. (ipod or i-pod).ti,ab. (287)
    56. mobile*.ti,ab. (84502)
    57. phone*.ti,ab. (30951)
    58. smartphone.ti,ab. (5396)
    59. technolog*.ti,ab. (394411)
    60. telephon*.ti,ab. (54456)
    61. wifi.ti,ab. (281)
    62. wireless.ti,ab. (11091)
    63. or/48-62 [HARDWARE] (817195)
    64. electronic mail/ (2459)
    65. text messaging/ (2040)
    66. exp videoconferencing/ (1572)
    67. exp internet/ (70489)
    68. mobile applications/ (3439)
    69. virtual reality/ (502)
    70. android.ti,ab. (1874)
    71. (app or apps).ti,ab. (22044)
    72. blog*.ti,ab. (1537)
    73. cyber*.ti,ab. (5586)
    74. (email* or e-mail*).ti,ab. (13513)
    75. facebook.ti,ab. (2501)
    76. instagram.ti,ab. (215)
    77. instant messag*.ti,ab. (247)
    78. internet*.ti,ab. (43734)
    79. media-based.ti,ab. (796)
    80. media-deliver*.ti,ab. (51)
    81. messag* service?.ti,ab. (1044)
    82. (multimedia or multi-media).ti,ab. (4808)
    83. new-media.ti,ab. (621)
    84. (online* or on-line*).ti,ab. (114701)
    85. podcast*.ti,ab. (618)
    86. reddit.ti,ab. (56)
    87. social network* site*.ti,ab. (944)
    88. sms.ti,ab. (4906)
    89. snapchat.ti,ab. (31)
    90. social-medi*.ti,ab. (9271)
    91. software.ti,ab. (138893)
    92. telecomm*.ti,ab. (3877)
    93. text-messag*.ti,ab. (3005)
    94. texting.ti,ab. (667)
    95. twitter.ti,ab. (2077)
    96. video-based.ti,ab. (1897)
    97. virtual*.ti,ab. (113968)
    98. vlog*.ti,ab. (29)
    99. web*.ti,ab. (125844)
    100. www.ti,ab. (1454)
    101. youtube.ti,ab. (1273)
    102. or/64-101 [SOFTWARE OR MEDIA] (565472)
    103. "Cell Phone Use"/ (56)
    104. 47 or 63 or 102 or 103 [ALL EHEALTH] (1310855)
    105. 34 and 104 [MSM AND EHEALTH] (5016)
    106. limit 105 to yr="1995 -Current" (4709)
    107. remove duplicates from 106 (4701)
