# Supplementary material for: Theories of change for e-health interventions targeting HIV/STIs and sexual risk, substance use and mental ill health amongst men who have sex with men: systematic review and synthesis
Source: Syst Rev. 2021 Jan 11;10:21. doi: 10.1186/s13643-020-01523-2 (PMC7798186; doi:10.1186/s13643-020-01523-2)
Supplement: Supplementary file 2 — Additional file 2. Individual and overarching theory of change logic models for the ‘Self-monitoring’ theory group. [file 13643_2020_1523_MOESM2_ESM.docx]

# Additional file 2. Individual and synthesised theory of change diagrams for the ‘Self-monitoring’ theory of change grouping

Figure 1. Theory of change diagram for TXT-Auto intervention^1^


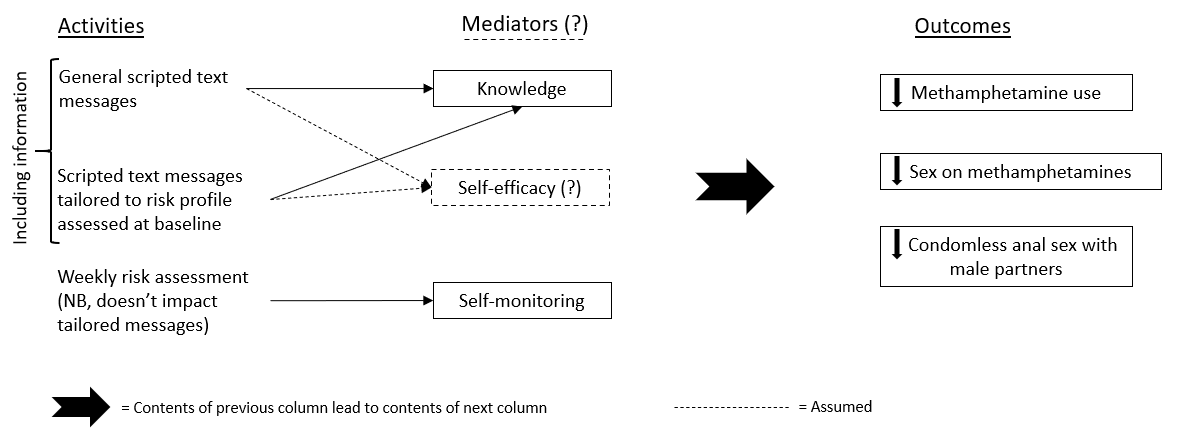


## Figure 2. Theory of change diagram for Smartphone Self-Monitoring intervention^2^


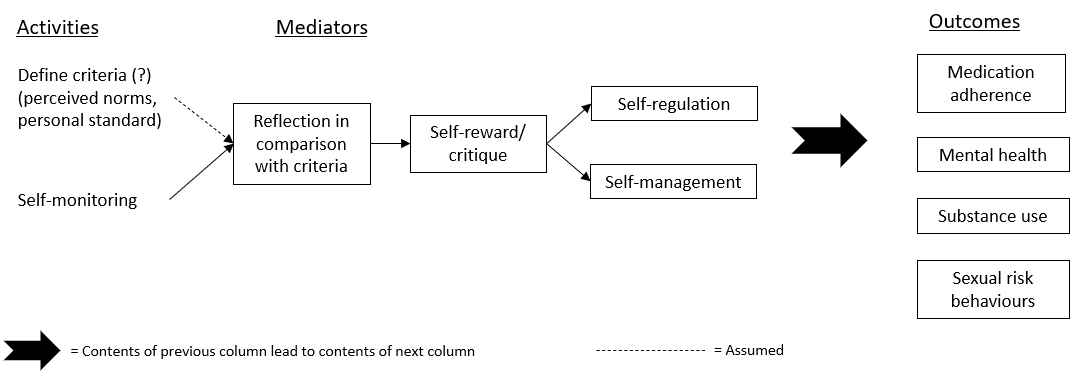


## Figure 3. ‘Self-monitoring’ synthesised theory of change diagram


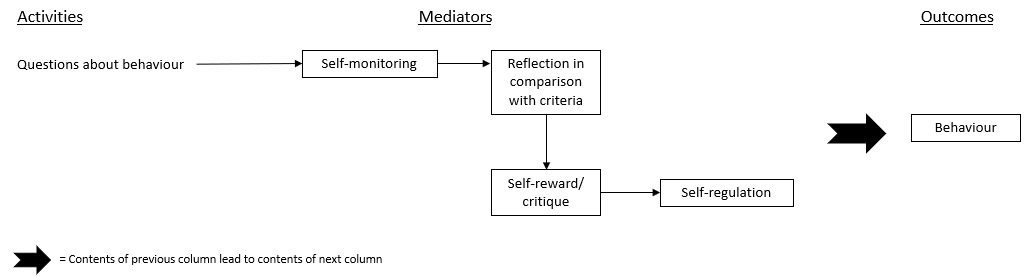


# References

1. Reback C, Fletcher J, Swendeman D, Metzner M. Theory‑Based Text‑Messaging to Reduce Methamphetamine Use and HIV Sexual Risk Behaviors Among Men Who Have Sex with Men: Automated Unidirectional Delivery Outperforms Bidirectional Peer Interactive Delivery. *AIDS and Behavior.* 2019;23(1):11.

2. Swendeman D, Ramanathan C, Baetscher L, et al. Smartphone self-monitoring to support self-management among people living with HIV: Perceived benefits and theory of change from a mixed-methods, randomized pilot study. *J Acquir Immune Defic Syndr.* 2015;69:12.
